# *COMBIT*: protocol of a randomised comparison trial of *COM*bined modified constraint induced movement therapy and *bi*manual intensive *t*raining with distributed model of standard upper limb rehabilitation in children with congenital hemiplegia

**DOI:** 10.1186/1471-2377-13-68

**Published:** 2013-06-28

**Authors:** Roslyn N Boyd, Jenny Ziviani, Leanne Sakzewski, Laura Miller, Joanne Bowden, Ross Cunnington, Robert Ware, Andrea Guzzetta, Richard AL Macdonell, Graeme D Jackson, David F Abbott, Stephen Rose

**Affiliations:** 1 Queensland Cerebral Palsy and Rehabilitation Research Centre, School of Medicine, Faculty of Health Sciences, The University of Queensland, Brisbane, Australia; 2 Children’s Allied Health Research, Royal Children’s Hospital Herston, Brisbane, Australia; 3 School of Health and Rehabilitation Sciences, The University of Queensland, Brisbane, Australia; 4 School of Psychology & Queensland Brain Institute, The University of Queensland, Brisbane, Australia; 5 CSIRO, ICT Australian e-Health Research Centre, Royal Brisbane and Women's Hospital, Centre for Clinical Research, The University of Queensland, Brisbane, Queensland, Australia; 6 Brain Research Institute, Florey Institute of Neuroscience and Mental Health (Melbourne Brain Centre, Austin Hospital), Victoria, Australia; 7 Queensland Children’s Medical Research Institute, The University of Queensland, Brisbane, Queensland, Australia; 8 School of Population Health, The University of Queensland, Brisbane, Australia; 9 Department of Developmental Neuroscience, Stella Maris Scientific Institute, Pisa, Italy; 10 Department of Medicine, The University of Melbourne, Victoria, Australia; 11 Department of Neurology, Austin Health, Heidelberg, Victoria, Australia; 12 Department of Radiology, The University of Melbourne, Victoria, Australia

**Keywords:** Congenital hemiplegia, Cerebral palsy, Stroke, Randomised clinical trial, Modified constraint induced movement therapy (mCIMT), Bimanual upper limb training, Hybrid constraint induced movement therapy (hCIMT), Functional magnetic resonance imaging (fMRI), Brain (re)organisation, International classification of functioning, Disability and health, Mastery motivation

## Abstract

**Introduction:**

Children with congenital hemiplegia often present with limitations in using their impaired upper limb which impacts on independence in activities of daily living, societal participation and quality of life. Traditional therapy has adopted a bimanual training approach (BIM) and more recently, modified constraint induced movement therapy (mCIMT) has emerged as a promising unimanual approach. Evidence of enhanced neuroplasticity following mCIMT suggests that the sequential application of mCIMT followed by bimanual training may optimise outcomes (Hybrid CIMT). It remains unclear whether more intensely delivered group based interventions (hCIMT) are superior to distributed models of individualised therapy. This study aims to determine the optimal density of upper limb training for children with congenital hemiplegia.

**Methods and analyses:**

A total of 50 children (25 in each group) with congenital hemiplegia will be recruited to participate in this randomized comparison trial. Children will be matched in pairs at baseline and randomly allocated to receive an intensive block group hybrid model of combined mCIMT followed by intensive bimanual training delivered in a day camp model (COMBiT; total dose 45 hours direct, 10 hours of indirect therapy), or a distributed model of standard occupational therapy and physiotherapy care (SC) over 12 weeks (total 45 hours direct and indirect therapy). Outcomes will be assessed at 13 weeks after commencement, and retention of effects tested at 26 weeks. The primary outcomes will be bimanual coordination and unimanual upper-limb capacity. Secondary outcomes will be participation and quality of life. Advanced brain imaging will assess neurovascular changes in response to treatment. Analysis will follow standard principles for RCTs, using two-group comparisons on all participants on an intention-to-treat basis. Comparisons will be between treatment groups using generalized linear models.

**Trial registration:**

ACTRN12613000181707

## Background

Congenital hemiplegia occurs in over one million children under 21 years of age in the industrialised world and is the most common type of cerebral palsy (CP), accounting for 36% diagnosed with this lifelong condition [1,2]. These children have limitations in their capacity to use their impaired upper limb and bimanual coordination deficits which impacts upon daily activities and participation in home, school and community life [3]. There are currently two diverse intensive therapy approaches aimed at improving upper limb performance. Traditional therapy has adopted a bimanual training approach (BIM) and more recently, constraint induced movement therapy (CIMT) has emerged as a promising unimanual approach.

Our 2009 published meta-analysis evaluating the efficacy of all non-surgical approaches to upper limb rehabilitation highlighted the absence of strong evidence for any particular model of therapy for improving upper limb outcomes in congenital hemiplegia [4]. There was consistent evidence for a small supplementary benefit for use of intramuscular Botulinum Toxin A (BoNT-A) injections combined with various models of upper limb training. No particular model of training, whether it was Neurodevelopmental Therapy (NDT), CIMT or Intensive Hand Arm Bimanual Training (HABIT) demonstrated a superior effect on activity limitations [4]. In the last three years, a significant number of additional randomised controlled trials, particularly of CIMT have been published [Sakzewski L, Ziviani J, Boyd RN: Efficacy of upper limb interventions for children with unilateral cerebral palsy: systematic review and meta-analysis update**,** submitted].

Classic CIMT involves application of a full arm cast to the unimpaired upper limb which is worn for 21 consecutive days and accompanied by six hours daily of upper limb training (total dose 126 hours) [5]. This model of therapy, initially used with adults post stroke, demonstrated significant improvements in upper limb function that were retained at 12 months post intervention [6]. The classic CIMT model has been used in a small number of studies for children with congenital hemiplegia demonstrating improved quality and amount of use of the impaired upper limb and acquisition of new upper limb motor skills [5,7]. A subsequent study compared the full classic CIMT protocol to half dose (60 hours) and demonstrated that for three to six year old children with congenital hemiplegia, 63 hours of training was sufficient to drive changes in upper limb function [5,8].

Classic CIMT has been criticised for not being child friendly or clinically feasible to implement. A significant number of modified CIMT (mCIMT) models have been developed. These have varied from intensive group based interventions [9-12], intensive individual therapy [13,14], distributed group or individual intervention [16-21] delivered either in the clinic [9,10], community [11], or in the home [14,15,17,20,21]. Despite the different modes of delivery and environmental context, findings consistently demonstrate mCIMT is superior to standard or usual care of a lesser dosage to improve quality and amount of use of the impaired upper limb [17,21], bimanual and unimanual efficiency of movement [17,21] and bimanual performance [16].

In contrast, when mCIMT has been compared to an equivalent dose of intensive bimanual training or goal directed occupational therapy, minimal differences have been found on most clinical measures, as both interventions yield similar improvements [10,11,13]. This highlights that the dose of therapy may be the critical ingredient rather than the type of therapy (e.g. unimanual versus bimanual).

Constraint induced movement therapy and modified protocols have a significant limitation, that is, they do not allow practice of bimanual tasks. Goals identified by children and their caregivers tend to be bimanual in nature [11,19]. This has led to the development of hybrid models of therapy that combine mCIMT and bimanual training. Two studies have investigated differing hybrid models of mCIMT followed by bimanual training. A long duration, distributed model of therapy performed over an eight week period (six weeks mCIMT 54 hours; two weeks bimanual 18 hrs) found significant gains in unimanual, bimanual and individualised outcomes compared to standard care for 2.5 to eight year old children with congenital hemiplegia [22]. A short duration model delivered over a three week period (two weeks mCIMT 30 hrs, one week bimanual 2.25 hrs), however was not superior to usual care to improve movement efficiency but did yield significant changes in self-care [23].

The differences in findings may reflect variations in dosage, the choice of outcome measures, and intensity or density of training. Recent classic CIMT studies have also acknowledged the need for bimanual training to follow CIMT and have developed a “transfer package” to address this requirement [7,24]. Further support for the idea of sequential application of interventions was obtained from examination of neuroplasticity findings with Transcranial Magnetic Stimulation (TMS). Children receiving mCIMT had greater and earlier use-dependent neuroplasticity than those receiving BIM (in a masked comparison) immediately post intervention which was sustained at six months [25]. This has important implications for the timing of interventions and the potential for optimizing the strengths of both by sequential application.

There is currently little evidence to support traditional standard care block models of upper limb therapy (e.g. six to eight weekly one hour sessions), as these alone are unlikely to provide an adequate dose to drive changes in upper limb function. Occupational therapy home programs have been shown to be effective in a high quality randomised controlled trial [26] and could allow an adequate dose of therapy to be achieved if accompanied with a distributed block model of intervention. As little as 15 minutes of home program practice four times per week over an eight week block of therapy was shown to be effective in improving functional goals and quality of upper limb skills (Quality of Upper Extremity Skills Test) [26]. It remains unclear whether a more intensive model of upper limb training is superior to a distributed standard care model augmented by a functionally focused home program to achieve a similar dosage of therapy to improve upper limb unimanual, bimanual and individualized outcomes.

This current study proposes a hybrid model which will sequentially combine modified constraint induced movement therapy (mCIMT) followed by intensive bimanual training (called COMBiT) and compare this to a standard upper limb training model in a randomised trial. We propose to compare the intensive group based COMBIT model (one week of 30 hours of mCIMT followed by one week of 30 hours of bimanual training of which 45 hours was direct and 10 hours of indirect upper limb training) to standard care delivered in an individualised and distributed model (standard occupational therapy (OT); total dose 45 hours direct and indirect over 12 weeks) for improving outcomes as they pertain to activity and participation for children with congenital hemiplegia. Standard care for children with congenital hemiplegia will be six weekly sessions of 1.5 hours of OT provided in an individualised format (9 hours direct) in combination with an individually designed home program delivered over a 12 week period (36 hours indirect training). Each approach, COMBiT or standard care may follow intramuscular BoNT-A injections to the forearm provided as part of standard clinical practice that is delivered to reduce forearm spasticity prior to upper limb training.

We intend to determine if COMBiT is effective in providing a superior and lasting benefit, compared to standard therapy. As therapy programs are resource intensive and time consuming it is important to determine if an intensive program such as COMBiT is superior to standard distributed models of conventional therapy for improved and sustained outcomes. If the effects of COMBiT are sustained and superior over a six month period this could offer a cost effective, timely model of care. This study has been funded by the National Health and Medical Research Council (NHMRC) of Australia (Project Grant 1003887).

## Methods and analyses

A matched pairs randomised comparison trial will be conducted to evaluate whether a novel rehabilitation model (COMBiT) will be more effective than conventional standard care (OT) for improving upper limb function in children with congenital hemiplegia.

The primary hypothesis to be tested is:

H^1^ COMBiT will reduce activity limitations (improve unimanual capacity and bimanual performance) to a greater extent than standard care.

The secondary hypotheses are:

H^2^ Use dependent neuroplasticity and neurovascular changes (fMRI) will be more extensive and retained for a longer duration in children undertaking COMBiT than those engaged in standard care.

H^3^ COMBiT will be more effective compared with standard care to increase participation and enhance quality of life.

Two broad aims will be addressed:

The primary aim of our study is to determine which model of upper limb training leads to greater changes in upper limb activity performance and whether changes are maintained to six months post intervention.

A secondary aim is to further our understanding of the central neurovascular mechanisms underlying changes in upper limb function according to the type of training applied. Improving our understanding of the mechanisms underpinning treatment efficacy is an essential next step towards providing effective treatment and sustained outcomes. In addition, understanding the nature and timing of the brain lesion and relationship to treatment response may assist with effective allocation of resources.

### Ethical considerations

Full ethical approval has been obtained by the Medical Ethics Committee of The University of Queensland (2011000553), The Royal Children’s Hospital Brisbane (HREC/11/QRCH/37) and The Cerebral Palsy League Ethics Committee (CPL-2012-004). Trial registration has been obtained with the Australian and New Zealand Clinical Trials Registry: (ACTRN12613000181707). Informed, written consent will be obtained from all parents or guardians and assent from participants (if 12 years of age or older) before entering the trial.

### Study sample and recruitment

Fifty children and youth with spastic hemiplegia aged 5–16 years will be recruited across Queensland, Australia. Potential study participants will be identified through a population-based research database, which currently comprises of over 1300 children with CP at the Queensland Cerebral Palsy and Rehabilitation Research Centre (QCPRRC), the Queensland Cerebral Palsy Register (QCPR), Queensland CP Health Service, Queensland Cerebral Palsy League and advertising to Occupational Therapists, Physiotherapists and Paediatricians at the Royal Children’s Hospital, Brisbane and in the community. The recruitment process will target both publicly funded services and private practitioners with the expectation that the sample will be representative of children with congenital hemiplegia.

### Inclusion criteria

The study will include school aged children and youth:

(1) With a confirmed diagnosis of congenital hemiplegia.

(2) Aged five to 16 years.

(3) Who have reduced upper limb function due to predominant spasticity rather than dystonia.

(4) Who can provide sufficient co-operation and cognitive understanding to participate in the therapy activities.

For a subset of children performing the Advanced Brain Imaging studies further inclusion criteria are:

i Sufficient co-operation to perform Advanced Brain Imaging studies for 45 minutes.

ii No exclusions for 3 Tesla Magnetic Resonance Imaging (3T MRI) including no metal implants, no shunts, no uncontrolled epilepsy as the later would be a confound.

### Exclusion criteria

Participants will be excluded if they have:

(1) Fixed contracture or severe muscle spasticity in the designated muscle groups.

(2) Previously undergone surgery in the upper limb (UL).

(3) Received BoNT-A injections within six weeks prior to baseline assessments.

### Sample size

The primary basis for sample size calculation for this study is adequate power for the primary hypothesis: H^1^ comparison between the functional effects of COMBiT compared to standard care at 26 weeks. Based on data from the previous study [25] a mean difference of 7 units (10% of the control group mean at baseline on the Melbourne Assessment of Unilateral Upper Limb Function (MUUL) is proposed as the minimum difference likely to have clinical implications [27,28]. We assume a standard deviation of 9 units. Based on a comparison with alpha = 0.05 and 80% power, 25 subjects in each group (n=50) are required.

For H^2^, our previous upper limb has shown 90% success on 3 repeated fMRI scans [25]. Using 3T fMRI we can see activation in the representative cortex for motor studies with good signal to noise ratio. We allow for some loss of information due to subject refusal (10%) and scans where motion is a confounder (10%). With 40 participants in an analysis of baseline to week 13 changes on fMRI, this study will have 80% power to detect a difference between groups of 0.9 SDs. If we consider the supplementary motor area (SMA) given coefficients of variation (COV) for control subjects performing motor tasks (COV of 11% in PM1 and 35% in SMA) [29] and activation signal of 1.5%, we are able to detect differences in percentage activation levels over time as small as 0.47.

### Randomisation

Following baseline assessments, children will be randomised within matched pairs to receive either COMBiT or standard care. To maximise homogeneity of the sample and minimise group differences at baseline, matching in pairs will be completed according to age (12 month bands), gender, and Manual Ability Classification System (MACS) level [30]. Treatment allocation will be recorded on a piece of folded paper inside a concealed opaque envelope. Using a sequence of computer-generated random numbers the number “1” or “2” will be allocated to each member of the pair. As each pair is randomised, they will be allocated the next consecutive envelope, which will be opened by non-study personnel who will read and record the treatment allocation. Study personnel will be informed of group allocation.

### Study procedure

**Figure 1:**
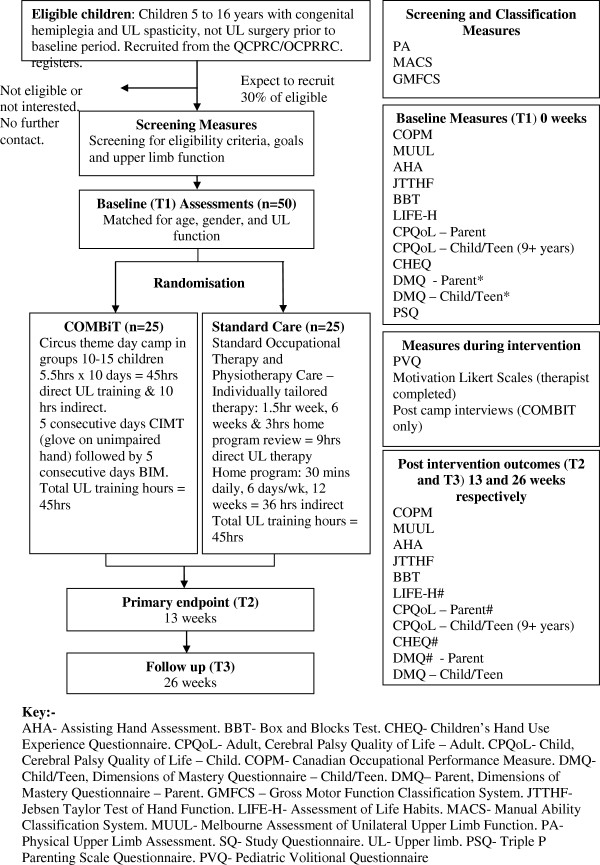
CONSORT flow chart for COMBiT trial.

Children will attend either the Royal Children’s Hospital or The University of Queensland in Brisbane for baseline and follow up assessments. Children randomised to COMBiT will participate in a two week day camp during school holidays commencing within one month of baseline assessments. Participants randomised to standard care will be allocated a local therapist and individual therapy sessions will commence within one month of baseline assessments. The experimental design and outcome measures are depicted in Figure 1.

### Study interventions

For the COMBiT day camp group, we plan to provide treatment in groups of 10–15 children over ten days. The initial five consecutive days will focus on mCIMT. After a weekend, BIM will be provided for the next five consecutive days. Therapy will comprise 45 hours direct upper limb training and 10 hours of indirect training in more general gross motor upper limb activities. COMBiT will have a novel circus theme. This intensive COMBiT therapy will be compared to standard care which involves individually tailored therapy in a distributed model for 1.5 hours per week for six weeks. This involves six hours individual therapy with OT/PT and three hours of home program demonstration (nine hours direct UL therapy). The home programs (completed by participants in their home/community environment) will comprise 30 minutes daily home practice six days/week for 12 weeks (36 hours indirect UL therapy). The COMBiT and the standard care programs will have the same dosages of upper limb therapy (COMBIT: 45 hours direct UL training; Standard care (SC): nine hours direct UL training and 36 hours indirect UL therapy). They will differ by (i) delivery method (block, group model versus distributed individualised model) (ii) varied construct (mCIMT versus goal directed training) and (iii) different environment.

#### ***(i) COMBIT: combined modified Constraint Induced Movement Therapy & Bimanual training***

In mCIMT, the unimpaired arm is constrained in an individually made glove and combined with intense practice of tasks to promote use of the impaired arm. In BIM, equal use of both hands is trained by engaging in bimanual tasks. During the mCIMT program, all therapeutic activities, circus activities, occupational tasks such as preparation and cleaning up of activities and all mealtimes will be performed predominantly with the impaired hand and the unimpaired hand will be constrained in a glove. In situations where the task demands use of two grasping hands, the participants will be required to co-operate with each other (in pairs) to complete the task with their impaired hands. This will be followed in the second week by an equal dose of BIM where bimanual use of both hands during all therapeutic activities, circus activities and occupational tasks will be required.

COMBiT will be conducted over two weeks of the school holidays in a “day camp model”. Participants will attend a community facility “Flipside Circus” for six hours a day, five days a week from 9am to 3pm. In total, 45 hours of the day camp program will directly target upper limb training, and 10 hours will provide indirect use of the UL in more general gross motor upper limb activities. For both mCIMT and BIM training, the content will include intensive activity based practice, circus activities as well as self-care and recreational activities. The group will be led and supervised closely by five to seven therapy staff to achieve a ratio of one trainer to two children. Five core occupational therapists and one physiotherapist will plan and lead all intervention groups. Daily grading of activities and modification of tasks for the participants in each group will be performed by the core therapists. Tasks and activities will focus on reducing upper limb activity limitations identified at baseline assessment and target three occupational performance goals identified by the parents and/or child. Planning activities for each group will require task analysis, and develop guidelines for grading to challenge children with varying capabilities. Specific details of the content of the COMBiT weekly program contents are presented in Additional file 1: Table S1. Prior to commencement of the daily program, staff will be briefed and given specific tasks with written instructions outlining how each activity will be performed for the specific children they are supervising. Professional circus trainers will lead the two hour circus workshops. The core therapy team will meet with the circus trainers to design these programs and at the end of each session to discuss and modify the program as required and provide guidance to grading of tasks for participants. A debriefing session for the entire COMBiT group with staff members and separately for the staff and volunteers will be conducted at the conclusion of each day. The core therapy team will also meet daily to review individual participants’ goals and continually grade their program. A daily record of attendance will be kept and anecdotal information from the day will also be recorded.

#### ***(ii) Standard care upper limb rehabilitation***

Standard care comprises an individualised and distributed model of occupational therapy upper limb training which consists of six sessions of 1.5 hours of individual direct therapy provided in a hospital or community setting over six weeks in combination with a 12 week home program. The weekly 1.5 hour sessions comprise one hour of direct therapy, and half an hour for home program development, demonstration and training with a total dose of nine hours of direct therapy. Families will be provided with a home program to practice goal areas for 12 weeks from the commencement of individual therapy sessions (30 minutes daily practice for six days/week for 12 weeks) for a total dose 36 hours of indirect UL therapy for the standard care group. The total anticipated therapy dose for standard care including direct therapy and home program practice is 45 hours of training.

### Concurrent therapies

During the study children will not be provided with any additional concomitant upper limb treatments, such as arm splinting, casting or additional upper limb training. Children will return to their regular therapy programs at the completion of the 13 week follow up assessments. Documentation of concurrent therapy programs (ongoing, additional therapy or interventions, change in spasticity medications or lower limb interventions) will be recorded at each follow up assessment as these would not be able to be controlled over a six month period.

#### ***Study duration***

Children will be recruited in blocks of 24 to 26 children at six monthly intervals to enable sessional testing of fMRI and training. The children will attend day camps during a holiday period. Recruitment will take 12 months, as suitable candidates could be drawn from our QCPRRC register (n=180 potential children). Potential participants can receive upper limb intramuscular BoNT-A injections prior to study entry as part of clinical practice. Entry into the study will be delayed for six weeks post injection for those whom have received intramuscular BoNT-A injections in the forearm to reduce spasticity.

### Measures

#### ***Classification measures***

**a) Manual Ability Classification system (MACs)** The MACS classifies the child’s ability to handle objects in daily activities on one of five levels [30]. The MACs has reported construct validity, and excellent inter-rater reliability (ICC=0.97 between therapists and 0.96 between therapists and parents) [30]. Children in the study will be MACS level I – III.

#### 

**b) Gross Motor Function Classification System (GMFCS)** The GMFCS classifies the child’s ability to carry out self-initiated movements related to sitting and walking across five levels [31]. The GMFCS has strong construct validity with the Gross Motor Function Measure (r=0.91) [32] and good inter observer reliability between professionals and between professionals and parents [33,34]. In this study sample of children with hemiplegia, it is expected that all children in the sample will be GMFCS level I or II.

#### 

**c) Zancolli scale** The Zancolli Scale [35] classifies severity of forearm alignment by measuring the contribution of spasticity and muscle length in the wrist and finger flexors in active wrist and finger extension. Three levels range from I (minimal flexion spasticity, complete extension of fingers with wrist in neutral position or less than twenty degrees of flexion) to III (severe flexion spasticity, no extension of fingers even with maximal wrist flexion). It is expected that participants will have either a Zancolli score level I or II.

#### 

**d) House functional classification scale** The House Functional Classification Scale [36] consists of nine grades ranging from 0 (does not use) to 8 (full spontaneous use) to rate functional use of the impaired upper limb.

#### 

**e) Classification of the brain lesion** The nature of the brain lesion will be classified using Magnetic Resonance Imaging (MRI) by a Child Neurologist. The classification system to be used is based on the presumed timing and nature of the insult that resulted in CP including both genetic and non-genetic aetiologies such as cortical malformations & hypoxic ischaemic injury [37,38]. Brain lesion severity will be assessed using a structured scoring proforma [39] based on the CH2 template [40], a highly detailed single-subject T1 template in MNI space, which is the international standard for brain mapping (International Consortium of Brain Mapping - ICBM). Lesions will be transcribed onto the proforma and the following measures obtained: number of (i) anatomical lobes involved, (ii) number of slices on the template that were impaired and (iii) size and distribution of the lesion measured by a total lesion score.

### ***Neurovascular measures***

#### 

**a) Whole-brain functional MRI studies** We have previously used functional Magnetic Resonance Imaging (fMRI) to examine functional reorganisation [41,42] and have combined this with TMS to localise the (re)organisation of the motor cortex in children with CP [25]. Functional MRI or blood oxygen level dependent (BOLD) contrast is a robust and non-invasive method of detection of regional tissue changes in venous oxygenation in response to task related activation [43].

Functional imaging will be performed at 3 Tesla on a Siemens MAGNETOM Trio MR scanner at the Centre for Advanced Imaging (CAI), at the University of Queensland. Use of 3T will reduce the time in the scanner for children and improve the resolution of data collected compared to 1.5T. To prepare for the real fMRI scan all children will practice in a mock MRI scanner using techniques that have achieved 90% compliance in our earlier studies [25]. During scanning of their anatomical images, the children will be able to watch a favourite video. Children will lie supine, with their head immobilised with an immobilisation pad to minimise head movement. In the scanner, children will perform two motor tasks, simple active wrist extension at 2 Hz and a complex motor task. These tasks are frequently impaired in children with hemiplegia and most likely to show a response to training. The motor paradigm will consist of a 2-condition block design, visually cued via instructions projected on a screen. The baseline condition is no movement. A recording of a metronome at 2 Hz will provide an auditory cue for the rate of movement. Verbal cues to commence and end the task will be given. The task and rest periods are 30 seconds with the activation cycle repeated four times.

Children with sufficient comprehension will also complete an additional complex motor task in the scanner. This is a timing versus sequencing task performed in a block design (two runs of six minutes each), where the subject alternates between a block of single index finger button pressing with a block of random sequences of 3-finger button presses. For the sequence task, visual cues of “123, 321, 213” numbers to denote a random sequence of pushing three buttons with their index, third and fourth fingers with their dominant hand. This complex task is designed to differentiate activation in the primary motor cortex and different aspects of the basal ganglia circuit. The rationale behind the simple and complex movement is based on previous studies that showed these movements are able to induce activation of the motor cortex and basal ganglia circuits [44]. Notably increased complexity of finger movements increases activation of the basal ganglia circuit, and thus provides an ideal model to utilise fMRI to locate function specific regions of the cortex associated with finger movements.

An additional five minutes of resting-state fMRI will also be collected for analysis of functional connectivity immediately after the motor tasks. Tasks performed prior to resting-state fMRI can influence functional connectivity [45], so the resting-state data will be collected after the motor paradigms have been completed to provide consistency. The whole assessment will take no longer than 50 minutes in the scanner. The actual movements performed in the scanner will be rated for speed, the range of motion actually performed, ability to isolate the movement and presence of mirror movements in the contralateral hand or general body movements for the active motor task and the number and timing on correct button sequences in the complex motor task.

Functional MRI will be acquired using a BOLD acquisition sequence (Gradient-recalled-echo (GRE) echo-planar imaging (EPI), Repetition Time (TR)=3.0s, Echo Time (TE)=30 ms, Flip angle = 85°, Slice thickness=3 mm, FOV=216 mm, 44 slices, 72 × 72 matrix yielding an in-plane resolution of 3.0 mm × 3.0 mm). A single set of T2-weighted anatomical, FLAIR and 3D T1 volumes will also be collected. Functional MRI image processing, analysis and visualisation will be performed using iBrain™ software [41], SPM8 analysis software (Wellcome Department of Imaging Neuroscience, London, UK) and the iBrain™ Analysis Toolbox for SPM [46].

Pre-processing of the fMRI images will include slice-timing correction using a temporal interpolation scheme to estimate the response at the time of commencement of each acquisition volume, motion correction (realignment) within session and nonlinear registration across sessions for each participant, and spatial normalisation to the standard Montreal Neurological Institute (MNI) template supplied with SPM. In the realignment step, images within a session will be aligned to a single target image within that time series to minimise the effects of participant motion between scans. The target selected by iBrain™ is the image whose within-brain centre-of-mass is located closest to the median of all images in that time series. Target images from each session of a subject will then be non-linearly spatially normalised to a subject-specific space in an iterative fashion in a manner similar to that described in Wilson & Abbott et al. [47] to ensure unbiased registration of images across sessions; this step is designed to correct, as far as practicable, non-linear image distortions that may differ from session to session. The step will be undertaken within subject rather than directly to the standard template to maximise the fidelity of within-subject registration. The mean of the within subject registered images will then be spatially normalised to the standard MNI template. Because many participants have large lesions, spatial normalisation to the MNI template will be undertaken using only an affine transform. In practice, the image transformations derived in each step described above will first be combined and then applied in one step to minimise resampling artefact when writing the final images. The spatially normalised image data will be smoothed with an isotropic Gaussian kernel at least twice the voxel size to fulfill the assumptions of Gaussian random field theory (RFT). Using generalized linear models (GLMs), statistical parametric maps will be computed for each session of each subject. Temporal autocorrelation will be modeled using a white noise and autoregressive AR(1) model within SPM. Motion correction parameters will be included as covariates of no interest. Details regarding the specific implementation of the GLM and RFT by SPM are available elsewhere [48]. Gross motion will be removed using scan-nulling regressors that account for all the variance in the motion-affected scan and the following three scans (to avoid possible T1 effects) [49]. Gross motion will be defined as motion exceeding 0.5 mm from one scan to the next, as heuristically this appears to sufficiently mitigate the confound in functional connectivity studies where the effect can be the most confounding [50]. Due to the heterogeneity in lesion location and size across participants, group analysis of intra-participant change in activation will be undertaken using a region of interest approach with the assistance of iBrain™ software. Laterality of regions will be quantitatively assessed using a method adapted from [51].

#### 

**b) Diffusion imaging acquisition and white matter fibre tracking** In addition to a number of standard radiological scans (T1, T2, FLAIR and 1 mm isotropic MPRAGE structural scan), diffusion-weighted images suitable for tractography studies will be acquired using a fully optimised single-shot, spin-echo echo-planar diffusion sequence. Diffusion weighted MRI data will be acquired to probe microstructural changes in white matter tracts delineated using tractography. The diffusion weighted data will be acquired using a 64-directional single-shot spin-echo echo-planar imaging sequence with the following imaging parameters: TR/TE 9500/116 ms; acquisition matrix 128 × 128 with field of view 30 × 30 cm (resulting in an in-plane resolution of 2.35 × 2.35 mm); 60 axial slices of thickness 2.5 mm; 64 non-collinear diffusion encoding directions; b-value 3000 s/mm2. The total imaging time for this sequence is 10 minutes. To reduce image distortions, an acceleration factor of 2 will be used, and a field map will be acquired to assist in the correction for residual distortions.

White matter fibre orientation distributions will be estimated using constrained spherical deconvolution [52] using MRtrix software [53], which allows the resolution of crossing fibres which is critically important for fibre-tracking [54]. Whole brain probabilistic tractography will be carried out using MRtrix. White matter pathways will be extracted using cortical and subcortical target regions identified in an automated fashion from high-resolution structural images using freesurfer (http://surfer.nmr.mgh.harvard.edu/). Alterations in white matter microstructure within these pathways will be assessed using traditional diffusion tensor imaging (DTI) metrics - including fractional anisotropy (FA) and mean diffusivity (MD) - as well as the novel apparent fibre density (AFD) measure [55] which takes into account the presence of crossing fibres. Tractography will be used to delineate individual pathways as described previously [56-58], and for connectivity-based parcellation [59,60]. Changes in white matter microstructure within delineated pathways following therapy will be assessed.

## Body functions and structures

The positive features of the upper motor neurone (UMN) syndrome will be measured at baseline to grade the severity of impairment in each group and to compare severity between the groups. Passive range of motion will be assessed primarily for the impaired shoulder, elbow, forearm, wrist flexors, fingers and thumb adductors using goniometry [61]. Spasticity will be measured using the Modified Tardieu Scale [62] at fast velocity in the forearm agonists and the Modified Ashworth Scale [63,64] in the same muscle groups.

The negative features of the UMN syndrome will be measured to describe the sample and to analyse their impact on outcome as a co-variate. Each test will be performed in both the impaired (hemiplegic limb) and unimpaired (hand writing limb) to compare sensory function between limbs. The following aspects of sensory impairment will be measured:

(i.) **Stereognosis**. will be assessed on the impaired and unimpaired limbs using the approach originally described by Feys [65]. Stereognosis was assessed according to a protocol using two sets of nine common objects, three familiar objects (key, spoon and peg) and six similar paired objects (button/coin, paperclip/safety pin and pen/pencil) [66]. With vision occluded, children will be presented with each item. A corresponding set of items will be used to allow children to identify the object in order to minimise any errors due to correct naming of the object. Scores ranged on a scale between 0–9, where participants scoring below 9/9 were considered to have impaired stereognosis [67].

(ii.) **Moving two point discrimination** (**M2PD**) will be measured using the Disk-criminator® (Baltimore, Maryland) on both the impaired and unimpaired limbs. Either one or two points will be randomly applied in continuous moving firm contact longitudinally to the pulp of the index finger with vision occluded [68]. The minimum distance participants can usually distinguish between two discrete points ranges from 2 mm (normal) to 15 mm (poor) [66,69].

(iii.) **Texture Tactile Perception** will be tested using the AsTex perspex board that displays tactile gratings of reducing tactile discrimination index [70]. Starting at the “rough” end of the board, movement of the child’s index finger, then thumb, then fifth finger will be guided by the examiner along the board at a constant speed in a standardized manner. Children will be instructed to stop immediately when the board feels smooth (gratings became too close together to determine their separation). Each point will be recorded, with the final outcome the average of three trials for each digit. The averaged scores will be converted to the tactile discrimination index for each finger using the chart available with the test kit.

(iv.) **Grip strength** will be measured using a hand held dynamometer (Smedley, Takei Scientific Instruments Co Ltd). Grip strength will be measured for the average of three attempts on the impaired and unimpaired limbs (kilograms force, Kgf) [71].

(v.) **Mirror movements** will be assessed and scored in each hand on the side of the body unintentionally performing the movement during three unimanual UL tasks: (i) rapid tapping of the index finger on the distal thumb, (ii) alternating supination and pronation of the forearm and (iii) repetitive alternate touching of each fingertip to the tip of the thumb of the same hand, in order. Participants will be videotaped and scored on a four point scale ranging from no clearly imitative movements to movement equal to that of the intended hand with a possible total score ranging from 0-12 [72].

## Primary outcomes

There will be a primary outcome measure for upper limb bimanual co-ordination (the Assisting Hand Assessment) and unimanual capacity (The Melbourne Assessment of Unilateral Upper limb Function).

a) Bimanual performance

(i) Assisting Hand Assessment (AHA)

The AHA is a Rasch analysed measure of bimanual hand performance [73]. The AHA is a performance measure and examines the effectiveness with which a child with a unilateral impairment spontaneously or typically uses their impaired hand in bimanual activities [73]. The test yields a range of scores between 22 and 88 which are subsequently scaled by transforming the total raw score to a percentage and range from 25 to 100. Conversion of these ordinal scores into logits (log odds probability units) which are equal interval measures is possible through Rasch analysis. Inter-rater and intra rater reliability is high for summed scores (ICC 0.98 and ICC 0.99 respectively). There are three versions of the AHA; small kids, school kids and an adolescent version. Test-retest reliability is high for small kids and school kids (ICC 0.99 and 0.98 respectively.) The AHA has demonstrated it is responsive to change in many clinical studies [16,22,24,74,75]. Reliability studies yielded a smallest detectable difference (SDD) of 3.89 raw scores for the small kids and 3.65 raw scores for the school kids version [76]. For this study, the AHA will be scored by two certified raters whom will be masked to group allocation and order of assessment. Scores will be transformed into logits for ease of interpretation.

b) Unimanual capacity

i. The Melbourne assessment of Unilateral Upper Limb function (MUUL)

The MUUL measures both upper limb impairment and quality of upper limb function [77]. It is designed for children aged 5–15 years with CP, and consists of sixteen criterion-referenced items measuring aspects of reach, grasp, release and manipulation. A set of scoring criteria for each item examines the quality of range of motion, accuracy, fluency and dexterity. The maximum possible raw score is 122, and raw scores are computed into percentage scores. Inter-rater and intra-rater reliability for the MUUL is very high for total test scores (ICC 0.95 and 0.97 respectively) and moderate to high for individual items (ICC 0.69 – 0.91). Test-retest reliability is high for total test scores and moderate to high for items [28]. The MUUL has good internal consistency (Cronbach’s alpha α=0.96) [28]. Construct and content validity for the MUUL was established during test development [77]. Previous results of a reliability study found a change of 12% for intra-rater reliability and 14% for inter-rater reliability was required to suggest a clinically significant effect [77]. More recent studies investigating reliability suggested the smallest detectible difference ranged from 7.4% [78] to 8.7% [79]. The MUUL has recently undergone Rasch analysis and unidimensionality of the scale was not confirmed [80]. Four distinct subscales were identified however, with only preliminarily evidence; it remains unclear whether any of the subscales will be better able to detect change following UL intervention [80]. Establishment of intra-rater reliability for the COMBiT study will be conducted to determine the SDD and define children who achieve a significant clinical response. The MUUL will be videotaped in accordance with the manual guidelines and will be later scored by an experienced occupational therapist masked to group allocation and order of assessment.

## Secondary outcomes

### ***Unimanual capacity***

i. Jebsen Taylor Test of Hand Function (JTTHF)

The JTTHF measures unilateral speed and dexterity on timed tasks [81]. The test measures speed and accuracy of performance on various complexities of grasp and release. The original test was designed and validated in adults and typically developing children and has been modified to exclude the writing task and reduced the maximum allowable time of each of the remaining six tasks to two minutes when utilised in children with congenital hemiplegia [25,75,82,83]. The JTTHF has been shown to be responsive to change due to an intervention [74]. There are concerns regarding the stability of test-retest performance in the unimpaired limb [74,75,82,83]. The test retest reliability and concurrent validity of the JTTHF with the Box and Blocks Test in children with congenital hemiplegia will be determined as part of the COMBiT study.

ii. Box and Blocks Test (BBT)

The BBT is a measure of gross manual dexterity [84]. It was initially developed specifically for use in adults with cerebral palsy the test comprises of a wooden box (53.7 cm × 25.4 cm) which is divided by a 15.2 cm high partition to form two equal compartments. 150 coloured wooden blocks all 2.5 cm in size are placed in one compartment. The participant is required to transfer as many blacks from one compartment to the other in 60 seconds. The score is the number of wooden blocks transferred in one minute [84,85]. Concurrent validity of the BBT is supported by a good correlation with the Minnesota Rate of Manipulation Test (r=0.91) and the General Aptitude Test Battery, Part 10 (r=.86). Inter rater reliability of the BBT was r = 1.00 and r = 0.999 for the right and left hands respectively. The reported test-retest reliability of the BBT (at six months) is ρ = 0.976 and 0.937 for right and left hands respectively [84]. Norms for 6–19 year olds are available [84].

## Bimanual performance

(i) Children’s Hand-Use Experience Questionnaire (CHEQ)

The CHEQ is a Rasch analysed questionnaire for children and youth (6 to 18 years) that examines their experience of using their impaired hand during bimanual activities [86]. Participants are required to answer twenty nine questions related to bimanual activities and identify their level of independence and whether one or two hands are used to complete the task. Three scales of perceived efficacy of grasp, time taken to complete the activity and degree of feeling of being bothered doing the activity are also rated for each of the bimanual activities. Summary scores for the questions can be generated, but for participants in a research trial, raw data will be transformed into logit scores by the test developers [86]. The CHEQ has not yet been used in clinical trials, and its sensitivity to change will be examined as part of the COMBiT study.

### ***Participation outcomes***

(i) Assessment of Life Habits (LIFE-H for children version 1.0)

The LIFE-H® [87] is designed for children aged 5 to 13 years and measures life habits in home, school and neighbourhood environments [87]. It is a questionnaire completed by the parent/caregiver about the child. The long form consists of 197 items divided into 12 categories and includes regular activities (eating meals, communication, and mobility) and social roles. A weighted score ranging from 0 to 10 is generated for each category and overall total. Construct validity was established during test development [87] and criterion validity with strong correlations between the LIFE-H and PEDI and Functional Independence Measure for Children (WeeFIM) are reported [88]. Adequate to excellent internal consistency (Cronbach’s α = 0.73 – 0.90 for categories, 0.97 for daily activities and 0.90 for social roles), intra-rater (ICC = 0.83 – 0.95 for daily activities), inter-rater (ICC = 0.8 – 0.91 for daily activities and 0.63 – 0.9 for social roles) and test-retest reliability (ICC = 0.73 for total score) have been established [89]. Four categories, reflecting the particular areas of difficulty in hand use and independence in daily life typically experienced by children with hemiplegia, will be evaluated in this study including nutrition (eg. mealtime activities), personal care (eg. dressing), education and recreation.

### ***Quality of life outcomes***

(i) The Cerebral Palsy Quality of Life questionnaire for children (CPQOL-child)

The CPQOL will be used to investigate quality of life from the perspectives of parents (CPQOL Primary Caregiver Questionnaire) and from the children themselves (children and youth of nine years or older- the CPQOL Child Report Questionnaire) [90]. The CPQOL-Child is a condition specific measure across seven broad domains of quality of life: social wellbeing and acceptance, functioning, participation and physical health, emotional wellbeing, access to services, pain and impact of disability and family health. Psychometric properties of the CPQOL are excellent with Cronbach’s α ranges from 0.74-0.92 (parent proxy report) and 0.80-0.90 (child self report) [91]. Adequate test re-test reliability (ICC = 0.76-0.89) is reported with moderate correlations with the CHQ, KIDSCREEN, and GMFCS. The CPQOL-teen version for youth 14–18 years has adequate correlations with a generic QOL instrument for both parent report (r = 0.40-0.46) and teen self report (r = 0.58-0.68) [92,93].

## Individualised occupational performance goals

(i) Canadian Occupational Performance Measure (COPM):

The COPM is a standardised individualised, client centred measure that evaluates client’s self-perception of occupational performance over time [94]. Participants identify areas of difficulty in everyday occupational performance across the domains of self-care, leisure and productivity and rate their performance and satisfaction for each problem on a scale from one to 10 [94]. An average score for performance and satisfaction is calculated. There is good evidence of construct, content and criterion validity [95-97]. The retest reliability of the performance and satisfaction scores on the COPM is high (ICC = 0.76-0.89) [98,99]. The COPM has demonstrated responsiveness to change in paediatric clinical trials [100,101] with a 2 point change on COPM performance reported as being clinically significant [94]. In the present study, the COPM will form the basis of goal setting for therapy. The COPM will be administered by one of the study occupational therapists. Children aged 8 years and older will be encouraged to identify three to five goals and rate their perceived level of performance and satisfaction on the 10-point scale in collaboration with their primary caregivers. For younger children or those with intellectual impairment, parent/caregivers will complete the COPM with input from their child.

## Environmental measures

(i) Study questionnaire

A study questionnaire has been developed to capture demographic information that has been shown in the literature to influence a child’s outcomes. Information will be collected on child’s age and gender, socio-economic status, family structure and supports, family income and current involvement in rehabilitation programs.

(ii) The Parenting Scale

The Parenting Scale [102] is a 30-item questionnaire measuring three dysfunctional parenting styles: laxness (permissive, inconsistent discipline); over-reactivity (harsh, emotional, authoritarian discipline and irritability); and verbosity (lengthy verbal responses) [103]. All 30 items are scored on a seven point scale, with low scores indicating good parenting practices and high scores indicating dysfunctional parenting. The Parenting Scale will be completed by the primary caregiver during baseline assessments and an overall measure of laxness, over reactivity and verbosity will be calculated. The total scaled score (the sum of all items divided by 30) will be used as the primary measure of parenting style in analyses. The Parenting Scale has demonstrated adequate internal consistency (α’s for subscales ranging from 0.78 to 0.85), good test-retest reliability (r = 0.84 for the total score), an ability to discriminate parents of clinic versus non-clinic children, and correlations with observed parenting style and child behaviour [102-104].

## Motivation

(i) The Dimensions of Mastery Questionnaire (DMQ)

The DMQ provides a primary caregiver’s and child’s self-perceptions of mastery motivation [105]. It consists of 45 items across seven subscales and two aspects of mastery motivation. Instrumental mastery focuses on persistence with tasks and includes the subscales of object-oriented persistence, gross motor persistence, social persistence with adults and social persistence with peers. Expressive mastery comprises subscales of negative reactions to failure and mastery pleasure. The final subscale, competence, is considered a separate construct which measures the child’s ability to master tasks relative to peers [105]. The DMQ takes approximately 15 minutes to complete and will be administered with both children and their primary caregivers according to standard administration procedures. This involves rating each of the 45 items on a five point scale ranging from 1=not at all typical to 5=very typical. Three is considered to be average for a typically developing child of the same age. Higher scores are considered to represent higher levels of motivation [105]. To assist with understanding in the younger age group (5 to 7 years) children may be prompted with ‘smiley faces’ representative of the scores 1=not at all typical through to 5=very typical. Seven individual subscale scores are calculated by summation of items in the subscale and dividing by the number of items to obtain an average score between 1 and 5. A total persistence score between 1 and 5 can be calculated from the average of the four instrumental subscales. A total mastery motivation score between 1 and 5 can also be calculated based on the average of the four instrumental subscales and the mastery pleasure subscale. The DMQ total motivation score (average of the four instrumental subscales and mastery pleasure) will be used as the primary measure of motivation as the DMQ total motivation score is representative of the child’s overall motivational predisposition including both instrumental and expressive mastery motivation. Individual DMQ subscale scores will be examined in secondary analyses. Test construction and clinical utility of the DMQ is satisfactory. The DMQ has high item internal consistency with Cronbach’s α‘s greater than 0.74 [105]. Test retest reliability for parents of preschool aged children is reported as high with ICC’s between 0.74 and 0.82 on instrumental subscales. Total score ICC’s are 0.76 with a standard error of measurement (SEM) of 7.31 [106]. Construct validity is strong with clear factorial evidence for scales on principal component analysis [106]. The psychometric properties and clinical utility of this measure have recently been evaluated as part of a systematic review of measures of motivation in school aged children with a physical disability or motor delay [107]. The test retest reliability of DMQ parent proxy report in school aged children will be investigated as part of this study.

(ii) The Pediatric Volitional Questionnaire (PVQ)

The PVQ is an observational assessment whereby the child’s volition is scored according to motivation and mastery behaviours observed during different activities [108]. This requires therapy sessions to be videotaped so that the child’s motivation and mastery behaviours when faced with therapy tasks of varying challenge can be observed. The PVQ has a four point scoring system where 1=passive through to 4=spontaneous. A mean level of volition ranging between 1 and 4 is calculated by summing all scores and dividing by the number of items (i.e. 14). The methodological quality of psychometric studies for the PVQ is poor based on very small samples sizes. Within these studies, the PVQ demonstrated adequate evidence for construct and criterion validity with Rasch analysis identifying good item spread (-0.96 to 1.34 logits) and no misfitting items [109]. Mean difficulty of items (0.00±0.66) and mean ability of participants (0.68±0.99) was well spread [109]. Reliability of the PVQ was weak with evidence of poor inter rater reliability (rater separation 4.11) [109]. For this project, the PVQ will be rated by the same rater. The psychometric properties and clinical utility of the PVQ measure will be evaluated as part of this study.

### ***Therapist observations and evaluation***

Therapist observations and evaluation of the child’s engagement, participation, persistence, task direction and task pleasure will occur during therapy sessions. This requires therapists to complete two Likert scales on task persistence and affect at the end of each therapy session. To assist in completing these scales the therapist will observe the following behaviours during sessions:

a. Persistence with a challenging problem, skill or task. Observation of the amount of task directed – is important in evaluating persistence. Greater persistence indicates greater mastery behaviour – which includes trying successfully or unsuccessfully to solve the problem or master the task motivation [110].

b. Embracing rather than avoiding challenge. Choosing challenging tasks in preference to easy ones indicates greater mastery motivation [110].

c. Displays of positive affect (smiling, pleasure, pride) associated with persistence. Positive displays of mastery (task) pleasure indicate higher levels of motivation [110].

d. Prematurely requesting help and avoiding challenges which indicate lower mastery motivation [110]

e. Any negative reactions and emotional responses to failure.

At the end of every therapy session therapists will evaluate the most common or predominant motivational behaviour observed. This will be measured on two scales:

i. *The Task Directed and Persistence Scale*: determines on a scale of 1 to 9 how task directed, persistent, goal focused and motivated a child was during the therapy sessions

ii. *The Child*’*s Affect Scale*: determines on a scale of 1 to 9 the level of positive affect demonstrated by a child during therapy sessions.

### ***Post intervention interviews***

The above measures will be supplemented with individual semi structured interviews post training to identify experiences of children participating in COMBiT, to gain insights into their experience of the day camp. Video footage of training sessions will be presented to facilitate interviews and discussions with children and their primary caregivers. Interviews will be videorecorded and transcribed verbatim.

## Blinding

Functional MRI data will be qualitatively analysed by neurologists masked to group allocation. Paediatric neurologists with fMRI training will independently rate scan quality (0–5), region of activation, change over time and patterns of reorganisation. Data for AHA and MUUL will be rated from videos masked to order and group allocation.

## Adverse events

Any minor and major events associated with intramuscular BoNT-A injections prior to the training study or due to either training model will be screened at 2 weeks by open-ended questions. If temporary weakness occurs following intramuscular BoNT-A injections, this can be addressed during the training programs. Any adverse events or unintended effects detected will be reviewed by a Rehabilitation Physician.

## Statistical analyses

Analysis will follow standard principles for RCTs, using two-group comparisons on all participants on an intention-to-treat basis. Primary analyses will include all evaluable data. Sensitivity analyses will use imputation techniques to account for potential bias as a consequence of non-ignorable missing data during follow-up. The primary comparison H^1^ at 6 months will be based on the AHA and MUUL scores and will be between treatment groups using generalized linear models, with terms included to account for matching and, potentially, confounding variables such as baseline unimanual capacity. Secondary analyses will use similar methods to compare the outcomes between groups for participation (domains of LIFE-H) and QOL (domains of CP-QOL). Where continuous data exhibit skewness not overcome by transformation, non-parametric methods (e.g. Mann–Whitney U Test) will be used for simple comparisons.

For H^2^: The magnitude of central neurovascular changes between groups will be determined using quantitative analysis of fMRI statistical parametric maps will be performed using *iBrain*™: regions of interest will be delineated for each individual (primary motor cortex PM1, supplementary motor area (SMA), and ipsilateral motor cortex (PM1ipsi) and active voxels in those regions will be counted. These data will be compared for each region over time using generalised estimating equations approach. In subjects where mirror movements did not occur, lateralisation between ipsilateral and contralateral PM1 will be assessed using an objective approach^CIC#188^ to determine the incidence and magnitude of brain reorganisation. Statistical significance will be at p<0.05.

## Discussion

This paper presents the background and design for a matched pairs randomized trial comparing an intensive block of combined mCIMT and BIM training (COMBIT) to a distributed standard care model of upper limb rehabilitation for children with congenital hemiplegia. To our knowledge this is the first study to directly compare the density of upper limb training, a block of intense COMBiT compared to an equal dose of a distributed model of individualised training. Furthermore, we will be evaluating the outcomes of the intervention program across all domains of the ICF using valid and reliable measurement tools.

## Abbreviations

CP: Cerebral palsy; CIMT: Constraint induced movement therapy; BIM: training Bimanual training; UL: Upper limb; MUUL: Melbourne Assessment of Unilateral Upper Limb Function; AHA: Assisting Hand Assessment; JTTHF: Jebsen Taylor Test of Hand Function; COPM: Canadian Occupational Performance Measure; LIFE-H: Assessment of Life Habits; CPQOL: Child Cerebral Palsy Quality of Life Questionnaire for Children; FMRI: Functional Magnetic Resonance Imaging; ICF: International Classification of Functioning, Disability and Health.

## Competing interests

The authors declare they have no competing interests.

## Authors’ contributions

RB is the chief investigator and together with JZ and LS designed and established this research study. RB, LS and JZ were responsible for the particular therapy contents. RB, LS, JZ and LM were responsible for ethics applications and reporting. RB, LS, LM, and JB were responsible for recruitment, data collection and implementation of the studies. DFA, RB, SR were responsible for the design, implementation, data collection, analysis of the Advanced Brain Imaging studies. RB, JZ, LS, will take lead roles on preparation of publications on the clinical outcomes of the study and RB, DFA, RC and SR will take lead roles on the neuroscience publications from the study. All authors have read and approved the final manuscript.

## Pre-publication history

The pre-publication history for this paper can be accessed here:

http://www.biomedcentral.com/1471-2377/13/68/prepub

## Supplementary Material

Additional file 1: Table S1 COMBIT weekly program example. Click here for file
