# Supplementary material for: COMBIT: protocol of a randomised comparison trial of COMbined modified constraint induced movement therapy and bimanual intensive training with distributed model of standard upper limb rehabilitation in children with congenital hemiplegia
Source: BMC Neurol. 2013 Jun 28;13:68. doi: 10.1186/1471-2377-13-68 (PMC3750247; doi:10.1186/1471-2377-13-68)
Supplement: Additional file 1: Table S1 — COMBIT weekly program example. [file 1471-2377-13-68-S1.docx]

**Table 1: COMBIT weekly program example**

### Week 1 - mCIMT

| **Session** | **Monday** | **Tuesday** | **Wednesday** | **Thursday** | **Friday** |
| --- | --- | --- | --- | --- | --- |
| 8:00 – 9:00 | Staff Briefing | Staff Briefing | Staff Briefing | Staff Briefing | Staff Briefing |
| 9.00 - 10.30 | Getting to know you –  Introduce gloves  Body tracing | Juggling balls  Decorating backdrop –  Edible playdoh | Craft: pasting, stickers  Cooking: decorate biscuits, smoothies | Ribbon stick making  Dressing up  Face painting | Craft: invitation making, back drop  Apple slinky |
| 10.30-11.00 | Morning Tea (buns with spread, squeeze jam/honey and fruit) | Morning Tea (edible playdoh, chopped fruit, toothpicks, spreading) | Morning Tea (biscuits, vege sticks, dips, smoothies) | Morning Tea (pikelets and cutters and rollers) | Morning Tea (apple slinky, cordial, yoghurt in bowls) |
| *11.00 - 1.00* | *Circus Skills* | *Circus Skills* | *Circus Skills* | *Circus Skills* | *Circus Skills* |
| 1:00 – 2:00 | Lunch (sandwiches, cookie cutters, chopped cheese and fruit )  washing up | Lunch (chicken nuggets and sauce, ham slices, vegie sticks)  washing up | Lunch (mini hot dogs, grated cheese, sauce, tinned fruit)  washing up | Lunch (create own sandwiches w fresh, or spread w help, chopped fruit) washing up | Lunch (crackers w toppings/spreads), cordial  washing up |
| 2:00 – 2:45 | Grassheads /Decorating  Individual goals | Team Olympics  Individual goals | Individual goals | Individual goals | Individual goals |
| 2:45 – 3:00 | Afternoon Tea (iceblocks, museli bars, water)  Debriefing – ball game | Afternoon Tea (Yoghurt fruit toobe, water)  Debriefing | Afternoon Tea (iceblocks, museli bars, water)  Debriefing | Afternoon Tea  (Cheese stick or fruit stick, water)  Debriefing | Afternoon Tea (biscuits from a bowl, popper)  Debriefing |
| 3:00 – 3:30 | Staff Debriefing | Staff Debriefing | Staff Debriefing | Staff Debriefing | Staff Debriefing |

**Week 2 - Bimanual**

| **Session** | **Monday** | **Tuesday** | **Wednesday** | **Thursday** | **Friday** |
| --- | --- | --- | --- | --- | --- |
| 8:00 – 9:00 | Staff Briefing | Staff Briefing | Staff Briefing | Staff Briefing | Staff Briefing |
| 9.00 - 10.30 | Reviewing the rules  Concert planning  Individual goals | Craft: Finishing invites (writing content)  Table top games (individual goals) | Craft activities  Face paint  Individual goals | Craft: Decorations for concert  Dressing up  (individual goals) | Concert Preparation: dressing up, face painting (individual goals) |
| 10.30-11.00 | Morning Tea (fruit skewers, saladas and spreads) | Morning Tea (individual yoghurt tubs, chopped fruit) | Morning Tea (faces: boxes of sultanas, pancakes, cordial) | Morning Tea (muesli bars, chopped not peeled fruit) | Morning Tea (saladas and spreads, cheese and tomato slices) |
| *11.00 - 1.00* | *Circus Skills* | *Circus Skills* | *Circus Skills* | *Circus Skills* | *CIRCUS CONCERT* |
| 1.00 – 2:00 | Lunch (wraps w meat, cheese and salad, chopped fruit)  washing up | Lunch (MYO salad w ham - grater, can opener, adults to chop)  washing up | Lunch (meat [patties to be chopped] and veg skewers, dips)  washing up | Lunch (pinwheel sandwiches w toothpicks, fruit)  washing up | Lunch party – bring a plate  washing up |
| 2:00 – 2:45 | ,  Cooking: Ginger balls/milo | Individual goals | Individual goals | Alkaseltzer rockets  Chocolate game | Piñata |
| 2:45 – 3:00 | Afternoon Tea (ginger balls and milo)  Debriefing | Afternoon Tea (zip lock bag w fruit + popper)  Debriefing | Afternoon Tea (tiny teddies/shapes, popper)  Debriefing | Afternoon Tea (le snack + popper)  Debriefing | Debriefing  Hand out certificates |
| 3:00 – 3:30 | Staff Debriefing | Staff Debriefing | Staff Debriefing | Staff Debriefing | Staff Debriefing |
